# Upper and Lower Limb Strength and Body Posture in Children with Congenital Hypothyroidism: An Observational Case-Control Study

**DOI:** 10.3390/ijerph17134830

**Published:** 2020-07-04

**Authors:** Jessica Brusa, Maria Cristina Maggio, Valerio Giustino, Ewan Thomas, Daniele Zangla, Angelo Iovane, Antonio Palma, Giovanni Corsello, Giuseppe Messina, Marianna Bellafiore

**Affiliations:** 1 Department of Psychology, Educational Science and Human Movement, University of Palermo, 90144 Palermo, Italy; brusajessica@gmail.com (J.B.); valerio.giustino@unipa.it (V.G.); ewan.thomas@unipa.it (E.T.); daniele.zangla@unipa.it (D.Z.); angelo.iovane@unipa.it (A.I.); antonio.palma@unipa.it (A.P.); marianna.bellafiore@unipa.it (M.B.); 2 Department of Health Promotion Sciences, Maternal and Infantile Care, Internal Medicine and Medical Specialities “G. D’Alessandro”, University of Palermo, 90127 Palermo, Italy; mariacristina.maggio@unipa.it (M.C.M.); giovanni.corsello@unipa.it (G.C.)

**Keywords:** congenital hypothyroidism, muscle strength, handgrip test, Sargent test, body sway, plantar pressure, posture

## Abstract

Background: Congenital hypothyroidism (CH) is an endocrine disease with a precocious significant impairment of growth and neuromotor development. Thyroid hormones are essential for central nervous system development, maturation, and myelination. Furthermore, thyroid hormone deficiency affects the function of several systems, including the musculoskeletal system. The disease has a significant incidence in the general population (1:3000–1:2000 newborns in Italy). The aim of the present study was to evaluate any differences in upper and lower limb strength, body sway, and plantar loading distribution in children with CH compared to healthy children. Methods: In this study, the case group was composed of children with CH (CHG), while the control group included healthy children (CG). Both groups comprised 19 children (CHG: female = 12; CG: female = 9). The maximum isometric handgrip strength and explosive-elastic lower limb strength were assessed with the handgrip test and the Sargent test, respectively. The stabilometric and baropodometric analyses were used to measure the Center of Pressure displacements and the plantar loading distribution between feet, respectively. The differences between groups were analyzed by a univariate analysis of covariance using as covariates weight and height with the significant level set at < 0.05. Results: We found that CHG children were shorter and thinner than CG ones (*p* < 0.05). No significant difference in the upper and lower limb strength was found between groups. CHG exhibited a significant greater Sway Path Length (*p* < 0.01) and Ellipse Surface (*p* < 0.05) than CG. Moreover, CHG displayed an asymmetric plantar loading distribution with a significant lower percentage in the right than in the left foot (*p* < 0.05). Moreover, a significant lower plantar loading percentage in the right foot of CHG than in the right foot of CG was observed (*p* < 0.05). Conclusions: These findings seem to suggest that CH does not affect muscle strength in early treated children. However, these patients show poor postural control ability and asymmetric plantar loading distribution. Increasing the physical activity in these children could improve their body posture.

## 1. Introduction

Hypothyroidism can occur prenatally (congenital) or after birth (acquired), and it is the most frequent endocrine disease in childhood [1,2,3]. Congenital hypothyroidism (CH) is a disease of the endocrine system caused by a thyroid hormone deficiency that induces a reduction in the body’s metabolic processes and affects the maturation of numerous tissues, including the central nervous system and musculoskeletal system [4]. Children affected by CH develop severe intellectual disability, with an intelligence quotient lower than 70. However, more than 95% of neonates with CH are oligosymptomatic or asymptomatic. Neonatal screening allows diagnosing the disease within the first days of life and to promptly treat these neonates, abating the risk of irreversible damage of growth and neurological development.

The thyroid gland produces triiodothyronine (T3) and thyroxine (T4) hormones, which are released into the blood, modulating multiple and coordinated activities and maintaining normal physiological functions of the body [5]. The main effects of these hormones are the increase of the basal metabolism and the speed of the energy expenditure useful for child’s growth [5,6,7].

In the last 20–30 years, the incidence of CH in Italy has increased, and the rate is estimated to be between 1:3000 and 1:2000 [8,9,10,11] newborns, with possible geographical differences secondary to the genetic background, different incidence of autoimmune thyroiditis between young adult women, and iodine deficiency. Lower TSH cut-off is the most important factor contributing to the increased number of CH diagnosis in Italy.

Patients are considered severe when congenital hypothyroidism have aplastic thyroid with severe hormone deficiency. These patients need higher doses of L-thyroxine.

CH is a multisystem disease with signs and symptoms that can have a negative impact on quality of life [12] including constraint in physical exercise and reduced physical performance [5,13,14].

Several studies reported that non-treated hypothyroidism in young patients showed impairments of the cardiovascular performance including myocardial contractility, pump function, heart rate, and hemodynamic parameters [5,14,15]. Furthermore, in non-treated patients, hypothyroidism affects many respiratory system aspects including control of breathing, control of ventilation [16], and other respiratory problems such as upper airway obstruction, pulmonary gas exchange, and exertional dyspnoea [17,18]. Concerning the musculoskeletal system, hypothyroidism in young patients showed a dysfunction related to muscle cell physiological processes such as protein synthesis, glycogen metabolism, and mitochondrial activity causing symptoms such as muscle weakness, muscle stiffness, and fatigue [3,19,20,21]. In particular, metabolic muscle deficit consists of a lower enzymatic activity involved both in the aerobic and in the anaerobic use of glucose [5].

Cardiovascular, respiratory, and musculoskeletal impairments in young subjects suffering by thyroid hormone disorders have been associated to a lower practice of physical activity than health peers and to a general reduction in their quality of life [5,22]. Indeed, this finding could lead to a sedentary lifestyle, weight gain, and low levels of health-related fitness including muscular strength [23].

MacFaul et al. reported that children and young patients with CH showed neurological disorders related to disorders of movement and posture, resulting in reduced motor control, poor coordination, impairment in gross and fine movements, body imbalance, and posture disorder [24]. Body posture is a feedback-feedforward system in which the central nervous system takes input signals coming from the various postural receptors, integrates and processes them, and transmits an efferent signal to the effector organs, i.e., the postural tonic system [25,26,27]. Changes in the musculoskeletal and neurological characteristics have been detected to alter body sway and postural balance and, moreover, plantar loading patterns [28,29].

Little is known about fitness characteristics and body posture in children with CH. Therefore, the aim of the present study was to assess features such as the muscle strength of the upper and lower limbs (i.e., maximum isometric handgrip strength and explosive-elastic lower limb strength), body balance, and plantar loading distribution between feet in children with CH and to compare them to healthy children. Our hypothesis is that young patients with CH show lower strength values and poorer levels of body posture.

## 2. Materials and Methods

### 2.1. Study Design

This is an observational case-control study, in which the case group included children affected by CH and was named the CH Group (CHG), while the control group comprised healthy children and was named the Control Group (CG). CHG children were recruited in the Pediatric Clinic of “ARNAS—G. Di Cristina” Children Hospital of Palermo, and data were collected from December 2017 to June 2018. CG children were recruited in several primary schools of Palermo from January 2018 to May 2018.

### 2.2. Participants

A number of 26 subjects for the CHG were initially recruited for the study but applying the exclusion criteria, only 19 participants (7 males and 12 females; age: 10.52 ± 3.03 years; height: 131.53 ± 15.22 cm; weight: 31.37 ± 10.8 kg; body mass index: 17.71 ± 3.54 kg/m^2^) were eligible for participating. All the patients of the CHG started the treatment with L-thyroxine within two weeks of age, and the dose/kg was 10–12 mcg/kg/day. They were euthyroid and showed normal levels of TSH, fT3, and fT4 at the time of the study. For the CG, we enrolled 19 children (10 males and 9 females; age: 10 ± 1.5 years; height: 142 ± 11.95 cm; weight: 39 ± 10.36 kg; body mass index: 19.09 ± 3.27 kg/m^2^). The exclusion criteria for participant selection for both groups were the following: physical injuries (*n* = 4 subjects excluded from the CHG) and neurological diseases (*n* = 2 subjects excluded from the CHG).

The study was approved by the Local Ethical Board of the University of Palermo and conformed to criteria for the use of persons in research as defined in the Declaration of Helsinki. Given that the participants were minors, parents or legal guardians provided their written informed consent to participate in this research after explaining to them the purpose of the study. All children participated voluntarily and could withdraw from the study at any time.

### 2.3. Assessment of Maximum Upper and Lower Limb Strength

The handgrip test was performed in order to measure the maximum isometric handgrip strength (MIHS), which is recognized as an important health indicator for determining musculoskeletal function, as well as weakness and disability [30]. Handgrip strength is a predictive factor for total muscle strength in healthy children, adolescents, and young adults [30].

Before the measurements, all the children performed a warm-up exercise to familiarize themselves with the mechanical dynamometer they would use and were encouraged to perform at maximum during the test. Each participant performed three trials of 3-s tests of the maximum isometric handgrip strength on a mechanical dynamometer (KernMap model 20K1—Kern^®^, Kern and Sohn GmbH, Balingen, Germany) sitting on a chair, at a 90° angle, with shoulder blades immobilized in the back, head in neutral position, looking forward, alternatively with the dominant hand and the non-dominant hand. A rest of 3 min was given between each trial. All measurements were performed by the same researcher and recorded in kg with a decimal point. The best performance of the three trials was considered for statistical analysis.

The vertical jump is a test that allows calculating the explosive-elastic lower limb strength [31]. In particular, jump height performance can be used as a variable to estimate lower limb power (equal to the muscle force multiplied by the velocity) [31,32,33]. We measured the maximum jump height using the Sargent test, which allows us to estimate the explosive-elastic lower limb strength (ELLS).

Firstly, participants were instructed to position themselves standing with one side facing the wall and feet together and to touch the wall as high as possible with the fingertips of the hand. Afterwards, in order to measure the maximum jump height, each participant, maintaining feet together, was asked to bend his knees and to perform an upward momentum, bringing the arms and hands extended upward, in order to touch the wall as high as possible during the jump. The same experimenter marked with a chalk the point on the wall reached by each participant with the fingertips before and during the jump. In order to measure the maximum jump height, the difference between the two points was calculated. Each participant performed three trials with a rest of 3 min between them using the best performance recorded for statistical analysis.

### 2.4. Stabilometric and Baropodometric Analysis

The stabilometric evaluation was performed using the FreeMed^®^ posturographic system (Sensor Medica^®^; Guidonia Montecelio, Rome, Italy), consisting of the freeMed^®^ Maxi platform and FreeStep^®^ software. The signal was digitized with a sampling frequency of 50 Hz. The used protocol was a sway test of 51.2 s of duration. In detail, each participant was required to stand barefoot in an orthostatic position on the platform with their head in a neutral position, looking forward, their arms along the trunk, their heels at a distance of 3 cm and their toes opened at a 30° angle [34]. The device measured the Center of Pressure (CoP) displacement and the variables examined were: Sway Path Length (SPL), Ellipse Surface (ES), Speed (S), amplitude of antero-posterior sway along the virtual y-axis (∆Y) and amplitude of medial-lateral sway along the virtual x-axis (∆X).

Baropodometric evaluation was carried out in an orthostatic position for 5 s on the same platform (freeMed^®^ Maxi; Sensor Medica^®^; Guidonia Montecelio, Rome, Italy) that measured the percentage of plantar loading distribution between feet.

### 2.5. Statistical Analysis

Data are presented as means ± standard deviations. A Shapiro–Wilk normality test was carried out to analyze the normality of distributions. The differences between groups were analyzed through univariate analysis of covariance. Covariates for all analysis were height and weight, in order to account for differences in anthropometric parameters across groups. The correlations between variables were assessed through Pearson’s correlation coefficients. IBM SPSS Statistics (Version 25) was used for all data analysis with the *p*-value considered significant when ˂0.05.

## 3. Results

As reported in Table 1, CHG children were shorter and thinner than CG ones (*p* < 0.05). No significant difference in age was found between both groups (*p* > 0.05).

Both groups showed a significant greater maximum isometric grip strength in the right hand compared with the left hand (*p* < 0.05), as reported in Table 2. However, we did not observe any significant difference in both hands between the groups, although the *p*-value was close to the statistical significance (*p* = 0.07). Moreover, explosive-elastic lower limb strength did not differ between the groups (Table 2).

As exhibited in Table 3, CHG showed a significant greater Sway Path Length (*p* < 0.01) and Ellipse Surface (*p* < 0.05) than CG. However, no significant difference was detected for Speed, ∆X, and ∆Y of CoP (*p* > 0.05).

**Figure 1:**
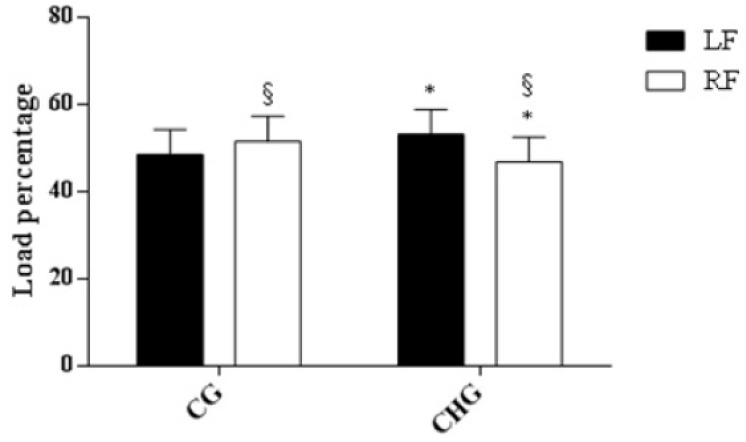
Percentage of plantar loading distribution. * § *p* < 0.05 Difference within group.

Baropodometry analysis (Figure 1) showed that the percentage of plantar loading distribution was significant greater in the left than in the right foot in CHG children (53.16 ± 5.67% versus 46.84 ± 5.67% respectively, *p* = 0.026), while in CG children, we found a greater plantar loading distribution in the right than in the left foot, although this difference was not significant (51.53 ± 5.77% versus 48.47 ± 5.78% respectively, *p* = 0.26). Moreover, we found that CHG presented a lower plantar loading percentage than CG in right foot (46.84 ± 5.67% versus 51.53 ± 5.78% respectively, *p* = 0.016).

Pearson’s analyses showed no correlation between all the variables of the assessments carried out in the study.

## 4. Discussion

The aim of our study was to evaluate any differences in upper and lower limb strength (i.e., maximum isometric handgrip strength and explosive-elastic lower limb strength), body sway, and plantar loading distribution in children with CH compared to healthy children.

Furthermore, although the main purpose of our study was not to compare the anthropometric characteristics of CH subjects with healthy ones, the statistical analysis showed significant lower values of weight and height for the CHG. These outcomes are in agreement with several studies reporting that children with hypothyroidism displayed a less advanced skeletal development and a delayed linear growth [35,36,37]; however, these data are in contrast with the expected outcome of growth in precociously well-treated patients, and there is the need to confirm by a study in a higher number of patients. A recent short review [6] stated that the aforementioned characteristics in childhood hypothyroidism are caused by defective endochondral ossification, which could lead to a complete stop in post-natal growth and skeletal dysplasia. The thyroid hormone replacement treatment is used in children to allow an adequate growth, although the achievement of a normal height in adult age may not always be reached because of many factors [36].

Based on our results, our hypothesis was partially confirmed. In fact, our findings showed no significant difference in the maximum isometric handgrip strength between groups, although the CHG showed a lower value for both hands compared to CG. Moreover, the assessment of the explosive-elastic lower limb strength also reported no difference between groups. Our findings suggested that there are no differences in two different types of strength in children’s limb (i.e., maximum isometric grip for upper limb and explosive-elastic for lower limb, respectively) between young patients with congenital hypothyroidism and healthy age-matched children. One of the possible key explanations for these outcomes can be the role played by the treatment. Although scientific evidence disagrees about the benefits of thyroid hormone replacement in intellectual and motor development in children with CH, it would appear that the timely intervention of levothyroxine replacement, an initial high-dose of this latter, and recurrent biochemical testing allow the achievement of normal cognitive and motor functioning levels [38,39]. Indeed, whether it is clearly recognized that untreated children with congenital hypothyroidism showed many development impairments (such as reduced intelligence quotient, behavior disorders, lesser fine and gross motor skill, lower verbal and arithmetic performance, vestibular and visuospatial impairment), and although cognitive and motor impairments may also be observed in subjects treated with levothyroxine, several researchers have demonstrated that the timing of treatment initiation and an adequate therapy ensure normal growth and development outcomes [38,39,40,41,42,43].

Another factor that can interpret our outcomes is the severity of congenital hypothyroidism. Indeed, since our sample was composed of children with non-severe CH, our findings are consistent with the study by Kempers et al. in which the authors reported that the severity of congenital hypothyroidism is one of the main aspects for long-term intellectual and motor impairment [44].

As concerns the within-group statistical analysis of muscle strength, we found a significantly greater maximum isometric grip strength in the right hand (i.e., dominant hand) compared with the left hand (i.e., non-dominant hand) for both groups. These results are in agreement with previously studies showing significant differences in children handgrip strength based on the hand dominance [45,46].

Regarding the postural outcomes, our sample reported the following features: (a) concerning the stabilometric analysis, CHG showed a significantly greater Sway Path Length and Ellipse Surface than CG; (b) regarding the baropodometric evaluation, CHG presented an asymmetric plantar loading distribution compared with CG, in which the plantar load was equally distributed. In particular, plantar loading percentage in the CHG right foot was lower than that in the CG right foot and CHG left foot. Several studies reported postural disturbance, including poor balance, in subjects affected by CH [44,47,48]. In agreement with a seminal study that investigated the neurological abnormalities in patients with CH, Macfaul et al. described disorders of movement and posture as common in these subjects [24]. We hypothesize that the reasons of these impairments could have a double explanation that involves the role of thyroid hormones both in the nervous system and in the muscular system. Indeed, as it is widely recognized that the cardiovascular and respiratory system benefit from the physiological amount and normal function of thyroid hormones [49], in the same way, due to a deficiency of these hormones, impairments in different regions of the brain or alterations in muscle functioning have been shown [4,24,49,50,51]. In particular, thyroid hormones are responsible for many nervous processes such as the terminal differentiation, the neuronal migration, and the myelination phenomena [4]. Frezzato et al. reported that a myelination deficiency has been showed in different nervous system regions that also may cause impairments in areas that manage the control, the planning, and the scheme of posture [4]. The automatic movements muscle, such as the muscle tone changes for controlling body posture, are managed by the extrapyramidal system and a lack in myelination in its nervous centers, and its associated pathways could negatively affect body posture features, including body sway [4,24].

As concerns the muscle alterations in patients with thyroid hormone deficiency, it is well known that these are fundamental for muscle fiber processes, such as protein synthesis, glycogen metabolism, and mitochondrial activity [49]. Moreover, regarding the musculature responsible for posture maintenance (i.e., the tonic postural system), many authors stated that thyroid hormone action affects the activities of mitochondrial enzymes mostly in type-I muscle fibers, whose distribution is greater in the muscles involved in maintaining body posture [50,51].

## 5. Conclusions

In conclusion, despite the main limitation of our study is the small sample recruited, our results seem to suggest that children with non-severe CH who are treated early achieve normal strength muscle levels; notwithstanding, they showed deficit in postural features. Although positive results have been found, very few scientific articles have investigated the effects of physical activity in hypothyroidism [52,53]. Hence, our future goal is to develop a protocol of experimental exercises to improve physical fitness in children with CH.

## Figures and Tables

**Table 1:** Description of participant’s anthropometric characteristics.

Participants	Age (Years)	Height (cm)	Weight (kg)	BMI (kg/m^2^)
CHG	10.52 ± 3.03	131.53 ± 15.22 *	31.37 ± 10.8 *	17.71 ± 3.54
CG	10 ± 1.5	142 ± 11.95	39 ± 10.36	19.09 ± 3.27

Legend. CHG: Congenital Hypothyroidism Group; CG: Control Group; * *p* < 0.05 CHG vs. CG.

**Table 2:** Assessment of maximal strength of upper and lower limb.

Variable	CHG	CG	*p*-Value
R-MIHS (kg)	10.78 ± 5.29 §	13.75 ± 4.65 *	0.074
L-MIHS (kg)	9.54 ± 4.56 §	12.07 ± 3.99 *	0.077
ELLS (cm)	16.26 ± 6.51	16.68 ± 5.64	0.83

Legend. R-MIHS: Right Maximum Isometric Handgrip Strength; L-MIHS: Left Maximum Isometric Handgrip Strength; ELLS: Explosive-Elastic Lower Limb Strength; CHG: Congenital Hypothyroidism Group; CG: Control Group; * § *p* < 0.05 Difference within group.

**Table 3:** Comparison of stabilometric parameters between CHG and CG children.

Parameter	CHG	CG	*p*-Value
SPL (mm)	846.84 ± 70.06	254.32 ± 81.94	0.01
ES (mm^2^)	532.8 ± 174.3	317.5 ± 331.7	0.016
Speed (mm/s)	18.97 ± 4.2	17.87 ± 6.38	0.55
∆X (mm)	21.25 ± 20.28	17.83 ± 10.93	0.89
∆Y (mm)	15.32 ± 12.06	20.14 ± 10.09	0.14

Legend. SPL: Sway Path Length; ES: Ellipse Surface; ∆X: amplitude of medial-lateral sway along the virtual x-axis; ∆Y: amplitude of antero-posterior sway along the virtual y-axis; CHG: Congenital hypothyroidism group; CG: Control Group.

## Abbreviations

CH: congenital hypothyroidism; T3: triiodothyronine; T4: thyroxine; TSH: Thyroid-Stimulating Hormone; CHG: Congenital Hypothyroidism Group; CG: Control Group; MIHS: maximum isometric handgrip strength; ELLS: explosive-elastic lower limb strength; CoP: Center of Pressure; SPL: Sway Path Length; ES: Ellipse Surface; S: Speed; ∆Y: amplitude of antero-posterior sway along the virtual y-axis; ∆X: amplitude of medial-lateral sway along the virtual x-axis.
